# Dynamics Relationship of Phyllosphere and Rhizosphere Bacterial Communities During the Development of *Bothriochloa ischaemum* in Copper Tailings

**DOI:** 10.3389/fmicb.2020.00869

**Published:** 2020-05-28

**Authors:** Tong Jia, Yushan Yao, Ruihong Wang, Tiehang Wu, Baofeng Chai

**Affiliations:** ^1^Shanxi Key Laboratory of Ecological Restoration on Loess Plateau, Institute of Loess Plateau, Shanxi University, Taiyuan, China; ^2^Department of Biology, Georgia Southern University, Statesboro, GA, United States

**Keywords:** *Bothriochloa ischaemum*, plant development, phyllosphere bacteria, rhizosphere bacteria, copper tailings dam

## Abstract

Copper mining and the byproducts associated with the industry have led to serious pollution in the Loess Plateau of China. There is a potential in improving the ecological restoration efficiency of such degraded land through combining microbial and plant remediation approaches. However, the community structure and function of phyllosphere and rhizosphere microorganisms and their response to plant development in copper tailings dams are poorly understood. This study investigated the impact of the phyllosphere and rhizosphere microbial communities on *Bothriochloa ischaemum* during three distinct plant development stages: seedling, tiller, and mature. The relative species abundance and Shannon index of bacterial communities of the rhizosphere during the seedling and tiller stages were distinct from that in the mature stage. Dominant bacteria at the level of phyla, such as *Proteobacteria*, *Cyanobacteria*, *Actinobacteria*, and *Bacteroidetes*, followed distinct patterns associated with plant development in the phyllosphere, but the predominant bacteria were similar in the rhizosphere. Redundancy analysis showed that aboveground total nitrogen and the carbon and nitrogen ratio of this plant species significantly affected phyllosphere bacterial community structure, whereas soil water content, soil nutrients, electrical conductivity, and salinity significantly affected rhizosphere bacterial community structure. Moreover, keystone phyllosphere and rhizosphere bacterial species differed significantly. This study sheds new light on understanding the dynamic relationship of phyllosphere and rhizosphere bacterial communities during plant development in copper tailings. These results are beneficial to the development and utilization of beneficial microbial communities at different stages of development, which might help to reclaim and stabilize tailings more effectively.

## Graphical Abstract

The characteristics of phyllosphere and rhizosphere bacterial communities during the development of *Bothriochloa ischaemum* in a copper tailings.

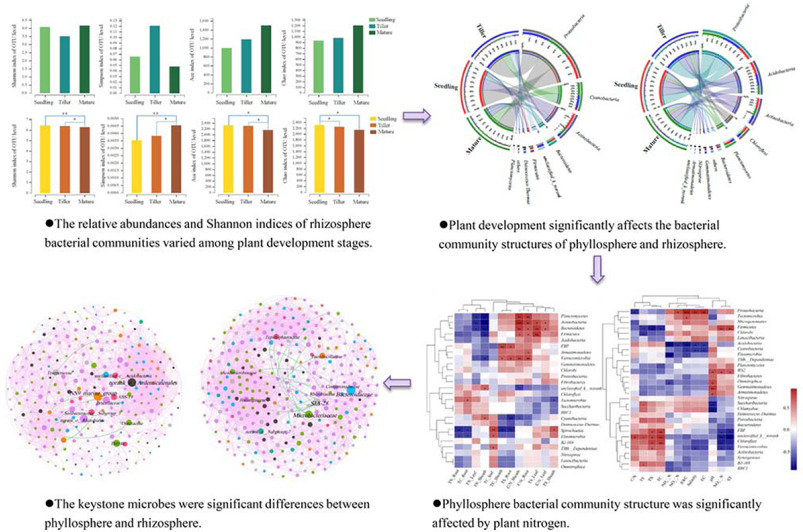

## Key Points:

(1) The phyllosphere bacterial community yields more unique OTUs than the rhizosphere community.
(2) Phyllosphere bacterial community structure is significantly affected by plant nitrogen content.
(3) Rhizosphere bacterial community structure is mainly affected by soil water content, soil nutrients, electrical conductivity, and salinity.
(4) Keystone phyllosphere and rhizosphere microbes significantly differ.

## Introduction

Soil contamination is a key issue of the mining industry. There are many metal mines in China which produce vast waste byproducts, consequently causing severe environmental pollution (Wong, 2003; Jia et al., 2017). The mutualistic symbiosis between microorganisms and plants promotes plant growth, aids in the absorption of water and nutrients, fortifies heavy metal resilience, and increases heavy metal absorption by plants while increasing overall plant biomass, thus improving the phytoremediation process (Lazaridou et al., 2011). Both plants and their associative microbial communities are able to remediate heavy metal contamination. Consequently, the application potential of combining both approaches to increase heavy metal absorption and transformation efficiency in contaminated soil is prodigious (Conesa et al., 2007; He et al., 2007).

The phyllosphere naturally contains a diverse and abundant community of non-pathogenic microbes (Vorholt, 2012). Conservative estimates indicate that 6.4 × 10^8^ km^2^ of global leaf surfaces harbor nearly 10^26^ bacteria, being the most bounteous and predominant colonists of the phyllosphere (Lindow and Brandl, 2003). The interaction between bacteria species and plants can be negative, causing disease, or positive, in that they can produce or modify hormones and metabolites or by interfering with pathogen growth. Their interactions can also be neutral and commensal (Lindow and Brandl, 2003). It has been shown that these bacterial populations are sufficiently large to play a dominant role in both vegetative growth (Chinnadurai et al., 2009; Janarthine and Eganathan, 2012) and vegetative resistance to pathogens (Costa et al., 2008; Balintkurti et al., 2010). However, to date most relevant research on phyllosphere bacteria has been concerned with the role of individual bacterium in protecting plants by suppressing pathogens (Costa et al., 2008) as well as how bacterial communities in the phyllosphere respond to climate change (Ren et al., 2014). Many factors control phyllosphere microbiota composition. For instance, environmental variables are dynamic, while host genotype and geographical location are more stable and constant (Vorholt, 2012). Although previous studies have identified certain factors (e.g., pH, heavy mental, and soil moisture) that have important impacts on structuring microbiota (Jia et al., 2018), results between studies still conflict.

Because root exudates are microbes’ primary source of nutrients, microbial interaction is particularly active in the rhizosphere, being the driver of both population density and activity (Raaijmakers et al., 2009; Bakker, 2012). Root-associated microbial communities are important in soil ecosystems, impacting plant growth and health, as well as influencing many soil biochemical processes (Zhao et al., 2015). Studies have shown that phyllosphere communities that reside on soil grown plants are similar to soil communities under natural conditions (Hardoim et al., 2012; Bodenhausen et al., 2013; Perazzolli et al., 2014).

Some plants including bean, soybean, maize, cabbage, cowpea, cotton, and *Arabidopsis* have exhibited age-related resistance (Marie-Pierre and Eric, 2010). Many studies have demonstrated that plant development can promote changes in microbial communities, but these studies have not determined which microbes or how such microbes contribute to observable changes. In some cases, host genotype is the primary factor that influences phyllosphere microbiome composition (Costa et al., 2012; Kim et al., 2012), although location has been shown in other cases to have the most significant impact on the composition of communities (Redford et al., 2010; Finkel et al., 2011; Rastogi et al., 2012). Season-dependent microbial communities that typically reside on perennial plants are similar from year to year (Redford and Fierer, 2009), while other microbial communities that reside on perennial plants change significantly, exhibiting significant seasonal variability (Jackson and Denney, 2011). Micallef et al. (2009) observed that rhizosphere microbial communities of *Arabidopsis* vary during the development of this plant species for which bulk density varies more significantly in communities during the early stages of plant development, decreasing as plants mature. Similarly, cultivar-dependent microbial communities have been shown to reside in the rhizosphere of young potato plants; however, such differences vanish as plants mature (Zgül et al., 2011).

Although many studies have shown that plant development affects plant associated microbial structures under natural conditions, there have only been limited studies conducted to date on the response of phyllosphere and rhizosphere bacterial communities to plant development in areas of environmental pollution. Moreover, a comprehensive comparison between phyllosphere and rhizosphere bacterial communities and their variable driving factors across plant development stages are extremely limited. Accordingly, this study investigated the structure and function of phyllosphere and rhizosphere bacterial communities associated with *Bothriochloa ischaemum* during three different plant development stages: seedling, tiller, and mature. The objectives of this study were (1) to survey phyllosphere and rhizosphere bacterial community composition during three distinct physiological developmental stages; (2) to examine the dynamic relationship of phyllosphere and rhizosphere bacterial communities during plant development; and (3) to test whether the driving factors of these phyllosphere and rhizosphere bacterial communities differ.

## Materials and Methods

### Site Description

Construction on the Shibahe tailings dam (latitude 35°15′∼35°17′ N, longitude 118°38′∼111°39′ E) commenced in 1969, and the dam is part of the Northern Copper Mine, situated in southern Shanxi Province, China. Currently, this copper tailings dam is comprised of 14 sub-dams. The study area is marked by four distinct seasons subject to a continental “monsoon” climate wherein annual mean temperature = 14°C, annual precipitation = ∼780 mm, and frost free days = greater than 200 d (Jia et al., 2017).

### Plant and Soil Sampling

We selected the No. 536 sub-dam of the Shibahe tailings dam in July 2017 for investigation. This sub-dam, in its twentieth year of restoration, was used for sampling during the three selected plant growth stages (seedling, tiller, and mature). The dominant plant species in this sub-dam was *B. ischaemum*. We randomly collected shoots and roots from *B. ischaemum* specimens as well as rhizosphere soil samples in three 1 m × 1 m sample plots, and each sample plot was spaced greater than 50 m apart. In each plot, 60 leaf samples were selected. The leaves were sealed in sterile plastic bags using tweezers sterilized in ethanol. For the plant samples, one subsample was used for physiochemical properties and the other was transported to the laboratory where it was stored (−20°C) in advance of high-throughput sequencing. For rhizosphere soil samples, visible roots and any residue were removed prior to homogenizing the soil fraction of each sample. Fresh soil samples were divided into two subsamples after sifting through a 2 mm sieve. The first subsample was stored (4°C) in advance of determining the physiochemical properties while the second was stored (−20°C) in advance of DNA extraction.

### Plant and Soil Chemical Properties

We measured the total carbon (TC), total nitrogen (TN), and total sulfur (TS) content of plant and soil samples using an elemental analyzer (vario EL/MACRO cube, Elementar, Hanau, Germany) (Tables 1, 2). Soil water (1:2.5 mass/volume) suspensions were shaken for 30 min in advance of measuring soil pH. Gravimetric analysis was used to measure soil moisture. We measured ammonium nitrogen (NH_4_^+^-N), nitrate nitrogen (NO_3_^+^-N), and nitrite nitrogen (NO_2_^+^-N) in soil using the Automatic Discrete Analyzer (CleverChem 380, DeChem-Tech, GmbH, Hamburg, Germany) (Table 2). The intra-group differences between the samples were smaller than the inter-group differences, which could reflect the overall situations of plant and soil.

**TABLE 1:** Mean values (±SE) of *Bothriochloa ischaemum* properties in copper tailings dam.

	**Seedling**	**Tiller**	**Mature**
TN_Leaf	3.213 ± 0.758^b^	93.748 ± 4.955^a^	5.199 ± 1.844^b^
TC_Leaf	47.158 ± 2.832^b^	45.319 ± 0.313^b^	78.431 ± 1.821^a^
C/N_Leaf	15.819 ± 2.462^a^	0.486 ± 0.028^b^	18.478 ± 4.912^a^
TS_Leaf	1.009 ± 0.702	0.157 ± 0.009	0.519 ± 0.184
TN_Sheath	0.817 ± 0.031^b^	41.381 ± 7.046^a^	1.329 ± 0.085^b^
TC_Sheath	42.720 ± 0.462^a^	33.346 ± 0.656^b^	6.631 ± 0.260^c^
C/N_Sheath	52.458 ± 2.341^a^	0.854 ± 0.148^c^	5.056 ± 0.537^b^
TS_Sheath	0.180 ± 0.022^b^	0.130 ± 0.008^b^	0.802 ± 0.093^a^
TN_Root	0.985 ± 0.016^b^	9.392 ± 3.120^a^	0.851 ± 0.035^b^
TC_Root	44.337 ± 0.032^b^	44.832 ± 0.096^a^	15.33 ± 0.277^c^
C/N_Root	45.038 ± 0.693^a^	6.949 ± 3.319^c^	18.089 ± 0.871^b^
TS_Root	0.627 ± 0.154^a^	0.244 ± 0.009^b^	0.032 ± 0.005^c^

*Significant differences between sites (Duncan test, *P* < 0.05) are denoted with letters (a > b > c). TN, total nitrogen; TC, total carbon; TS, total sulfur; C/N, the ratio of carbon and nitrogen; TC, the unit of TN; TS, g⋅kg^–1^.*

**TABLE 2:** Soil chemical properties of copper tailings dam.

	**Seedling**	**Tiller**	**Mature**
SWC (%)	0.841 ± 0.036^c^	15.499 ± 1.260^b^	86.162 ± 0.213^a^
pH	8.203 ± 0.020^b^	8.913 ± 0.019^a^	8.113 ± 0.029^c^
NH_4_^+^-N (mg⋅kg^–1^)	0.156 ± 0.029*bc*	0.273 ± 0.116*ab*	0.427 ± 0.075^a^
NO_3_^+^-N (mg⋅kg^–1^)	0.064 ± 0.006^c^	0.220 ± 0.019^a^	0.134 ± 0.007^b^
NO_2_^+^-N (mg⋅kg^–1^)	0.006 ± 0.001^b^	0.008 ± 0.001^b^	0.011 ± 0.002^a^
TN (%)	0.082 ± 0.001^a^	0.029 ± 0.003^c^	0.047 ± 0.005^b^
TC (%)	6.017 ± 0.919^a^	0.627 ± 0.018^b^	1.033 ± 0.041^b^
C/N	73.516 ± 11.817^a^	21.681 ± 1.164^b^	22.058 ± 1.474^b^
TS (%)	0.459 ± 0.294^a^	0.065 ± 0.002^b^	0.144 ± 0.017*ab*
ST (°C)	23.900 ± 0.473^c^	28.833 ± 0.698^a^	26.867 ± 0.484^b^
Salinity (mg⋅L^–1^)	0.000 ± 0.000^c^	14.333 ± 3.844^b^	31.667 ± 1.202^a^
EC (μs⋅cm^–1^)	0.000 ± 0.000^c^	26.667 ± 7.219^b^	57.667 ± 1.667^a^

*Values represent mean with standard error in parenthesis. Significant differences between sites (Duncan test, *P* < 0.05) are denoted with letters (a > b > c). SWC, soil water content; NH_4_^+^-N, ammonium nitrogen; NO_3_^+^-N, nitrate nitrogen; NO_2_^+^-N, nitrite nitrogen; TN, total nitrogen; TC, total carbon; TS, total sulfur; C/N, the ratio of carbon and nitrogen; ST, soil temperature; EC, electrical conductivity.*

### DNA Extraction, PCR Amplification, and Miseq Sequencing

Thirty leaf samples were randomly collected from each plot. These leaf samples were washed three times in a sterile phosphate buffer solution (PBS: NaCl, KCl, Na_2_HPO_4_, and KH_2_PO_4_) before being filtered through a sterile membrane filter (0.2 μm pore size) (Millipore, Jinteng, Tianjin, China). These filtered samples used to extract microbial DNA were sealed in sterile centrifuge tubes. The EZNA^®^ Soil DNA Kit (Omega Bio-tek, Norcross, GA, United States) was used to extract plant and soil microbial DNA under the manufacturer’s protocol. Extracted DNA was quantified using a NanoDrop ND-1000 UV-Vis Spectrophotometer (NanoDrop Technologies, Wilmington, DE, United States). Amplification of the V4-V5 hyper variable region of the 16S rRNA bacterial gene was conducted using primers 515F (5′-GTGCCAGCMGCCGCGG-3′) and 907R (5′- CCGTCAATTCMTTTRAGT TT-3′). Sequencing was conducted at Shanghai Majorbio Bio-pharm Technology (Shanghai, China) using the MiSeq platform (Illumina, Inc., United States). The bacterial sequences have been deposited in the SRA of the NCBI database under the SRA accession: PRJNA600330.

### Statistical Analysis

SPSS 20 was used to calculate the data that we derived from the aforementioned analyses, while Circos software was used to build the Circos graph (Krzywinski and Schein, 2009). Heatmapping of the top ten genera in each sample was conducted using the R packages. Principal component analysis (PCA) and redundancy analysis (RDA) were used to analyze relationships between bacteria and environmental factors using Canoco 5.0 (Microcomputer Power, United States). We used one-way analysis of variance (ANOVA) to determine the differences in environmental parameters, alpha diversity (α-diversity) indices, and the relative species abundance of the dominant bacterial species among the three selected plant growth stages. Additionally, we used the Mantel test to determine correlations between bacterial communities and environmental variables. We used the interactive platform Gephi to explore and visualize networks (Bastian et al., 2009). Structural equation models (SEM) was analyzed with AMOS 13.0.

## Results

### Overall Taxonomic Distribution and Bacterial Diversity

**FIGURE 1:**
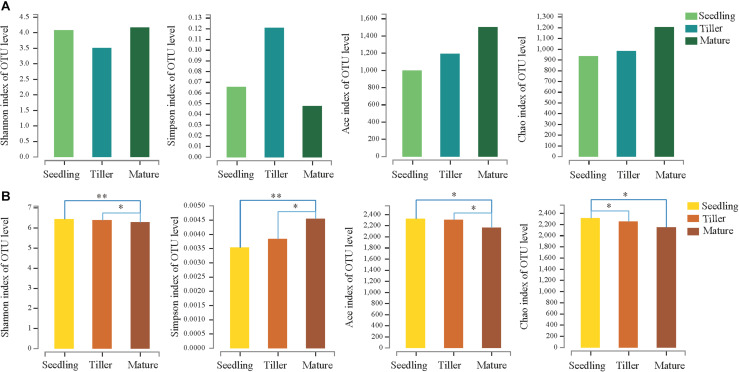
Estimated values of phyllosphere **(A)** and rhizosphere **(B)** bacterial community relative abundances and diversity index. Asterisks indicate significant differences among plant development stages, ^∗∗^*P* < 0.01, ^∗^*P* < 0.05.

We obtained a total of 705,846 high-quality sequence reads of an average length of 396 bp across all samples. A total of 1,982 bacterial OTUs from phyllosphere and 2,909 bacterial OTUs from rhizosphere soil samples were determined based on 97% sequence similarity. Taxonomically classified OTUs were all associated with 32 phyla, 80 classes, 70 orders, 319 families, and 563 genera in rhizosphere soil samples. For phyllosphere samples, taxonomically classified OTUs were associated with 26 phyla, 59 classes, 140 orders, 287 families, and 589 genera. The Venn diagram revealed that 480 OTUs were common to all phyllosphere bacterial communities while 1,837 were common to all rhizosphere bacterial communities (Supplementary Figure S1). Phyllosphere bacterial communities yielded a greater number of unique OTUs compared to rhizosphere bacterial communities during each plant development stage (Supplementary Figure S1). The relative abundance (Ace and Chao1) and diversity (Shannon and Simpson) index values of the phyllosphere and rhizosphere bacterial communities revealed the occurrence of dynamic change during the three plant development stages (Figure 1).

### Comparison Between Bacterial Communities Among Plant Development Stages

**FIGURE 2:**
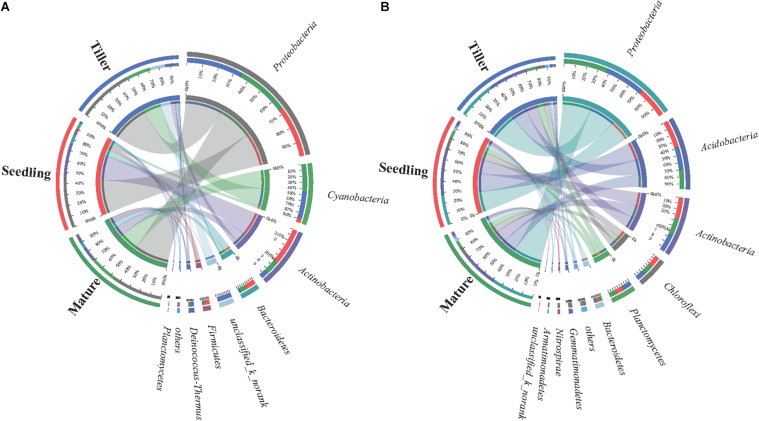
Distribution of phyllosphere **(A)** and rhizosphere **(B)** bacterial communities for each sample at phylum level. The width of the bars from each phylum indicate the relative abundance of that phylum in the sample.

The dominant bacterial species in samples from the phyllosphere were *Proteobacteria*, *Cyanobacteria*, *Actinobacteria*, and *Bacteroidetes* (Supplementary Figure S2A). *Cyanobacteria* increased from 6.9% (seedling) to 47% (mature), and *Actinobacteria* decreased from 63% (seedling) to 11% (tiller) (Figure 2A). The five prevailing bacterial phyla found in rhizosphere samples were *Proteobacteria*, *Acidobacteria*, *Actinobacteria*, *Chloroflexi*, and *Planctomycetes*, and they were present in greater than 90% of bacterial sequences during plant development stages (Figure 2B). Additionally, there was an increase in the relative abundance of *Proteobacteria* along with plant development in rhizosphere samples, while the relative abundance decreased in *Acidobacteria* as plant development continued to progress.

The Bray-Curtis distance analysis based on PCA and ANOSIM was conducted to determine the dissimilarity of bacterial communities among each plant development stage (Supplementary Figure S3). The results indicated that the first two axes (PC1 and PC2) explained 40.74 and 42.19%, respectively, of total variation in phyllosphere and rhizosphere bacteria communities during three plant development stages. ANOSIM revealed significant differences in both phyllosphere (Bray–Curtis ANOSIM = 0.885; *P* = 0.003) and rhizosphere (Bray–Curtis ANOSIM = 0.893; *P* = 0.006) bacterial community structure among the different plant growth stages.

**FIGURE 3:**
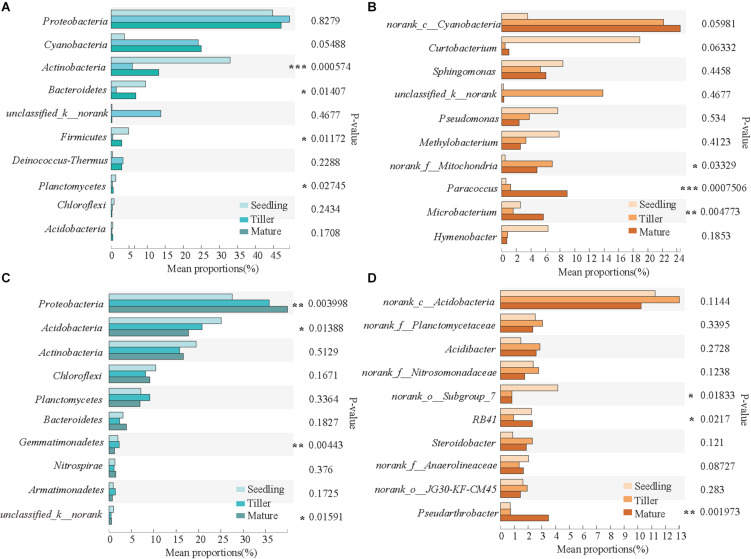
Relative abundances of top 10 bacterial phyla and genera that showed significant differences among phyllosphere **(A,B)** and rhizosphere **(C,D)** samples from the seedling, tiller, and mature stages. A one-way ANOVA was used to evaluate the significance of differences between the indicated groups. *P < 0.05; **P < 0.01; and ***P < 0.001.

The top 10 dominant phyla and genera are shown in Figure 3. For the phyllosphere bacterial community, significant differences were found in *Actinobacteria*, *Bacteroidetes*, *Firmicutes*, and *Planctomycetes* at a phylum level, and significant differences were found in *Paracoccus*, *Microbacterium*, and *norank_f_Mitochondria* at a genus level among the plant development stages (Figures 3A,B). For the rhizosphere bacterial community, *Proteobacteria*, *Acidobacteria*, *Gemmatimonadetes*, and *unclassified_k__norank* exhibited significant differences at a phylum level, and *norank_o_Subgroup_7*, *RB41*, and *Pseudarthrobacter* were distinct at a genus level among the plant development stages (Figures 3C,D).

### Relationship Between Bacterial Community Structure and Plant and Soil Characteristics

**FIGURE 4:**
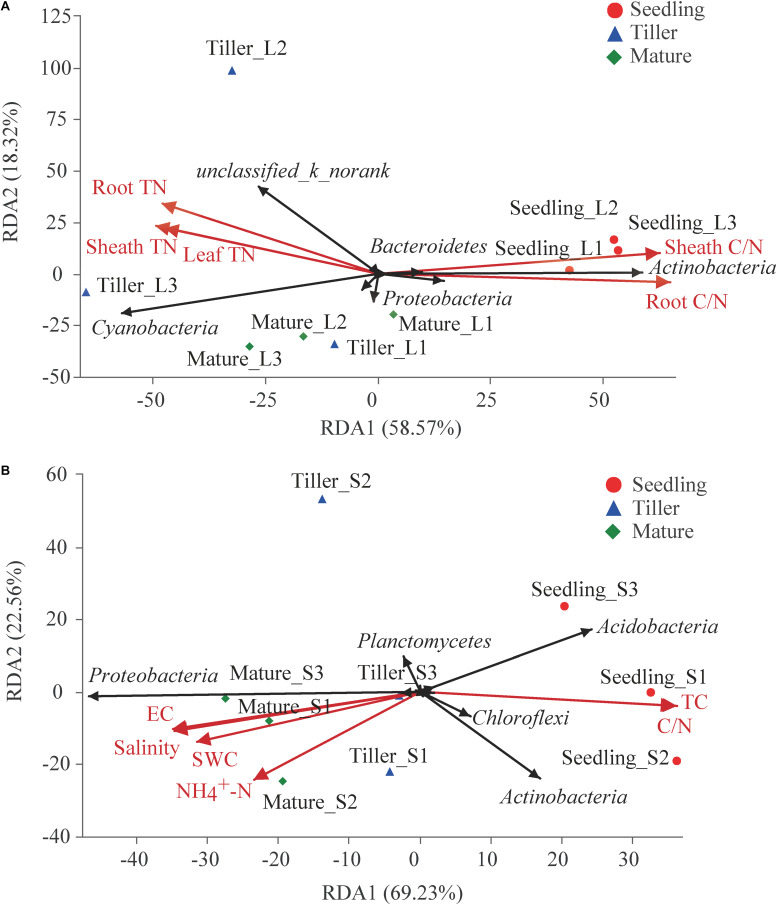
Redundancy analysis (RDA) analysis of the top 10 bacterial phyla and leaf or soil characteristics. Phyllosphere and rhizosphere bacterial communities are shown in **(A,B)**, respectively. The values of axes 1 and 2 are the percentages explained by the corresponding axis.

The experiment evaluated the effect of these ecological factors on the top 10 bacterial phyla in phyllosphere and rhizosphere communities using RDA and found a correlation among plant and soil bacterial community structure and characteristics (Figure 4). Results showed that 76.89% of variation in phyllosphere bacteria could be explained by leaf properties (Figure 4A). Phyllosphere bacterial community structure was significantly affected by plant N (including leaf TN, sheath TN, and root TN) as well as the C/N of leaves and sheaths (Figure 4A). Soil properties could explain 91.79% variability in rhizosphere bacterial community structure (Figure 4B). Six soil characteristics were chosen for RDA after removing redundant variables. As shown in Figure 4B, soil water content (SWC) (*P* = 0.015), soil TC (*P* = 0.005), NO_4_^+^-N (*P* = 0.028), C/N (*P* = 0.003), electrical conductivity (EC) (*P* = 0.002), and salinity (*P* = 0.002) significantly affected the bacterial community structure of the rhizosphere. Both the physiological properties of plant species and soil properties had significant effects on phyllosphere (Mantel test, *P* = 0.008 and *r* = 0.531) and rhizosphere (Mantel test, *P* = 0.001 and *r* = 0.658) bacterial communities, respectively.

**FIGURE 5:**
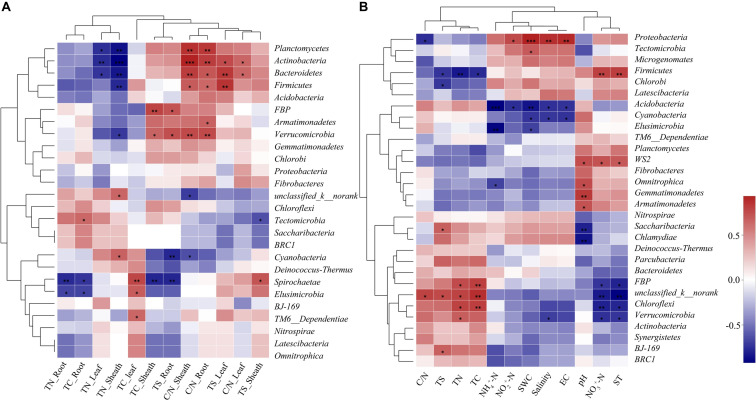
Correlation heat map of the top thirty phyla and soil properties and vegetation. X and Y axis are environmental factors and phyla. R in different colors to show, the right side of the legend is the color range of different *r* values. *P < 0.05; **P < 0.01; and ***P < 0.001. Phyllosphere and rhizosphere bacterial communities are shown in **(A, B)**, respectively.

**FIGURE 6:**
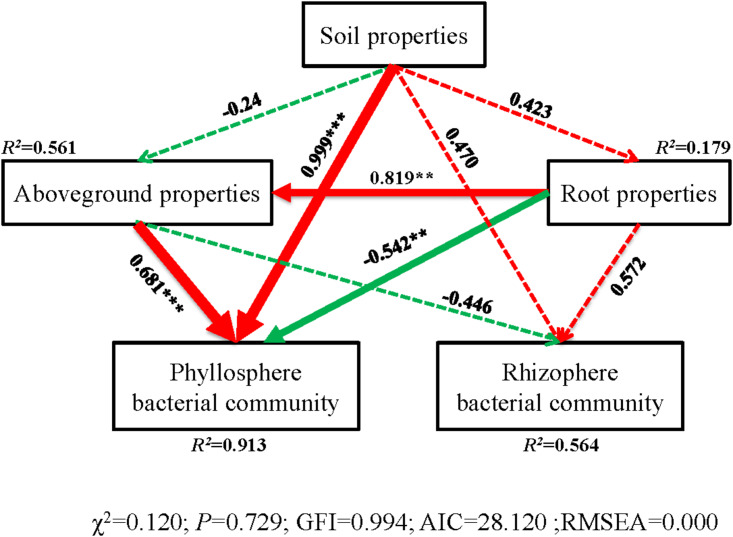
Structural equation model (SEM) illustrating the effects of soil properties on physicochemical characteristics (aboveground and root) and bacterial communities of phyllosphere and rhizophere. Continuous and dashed arrows represent the significant and non-significant relationships, respectively. Adjacent number that are labeled in the same direction as the arrow represents path coefficients, and the width of the arrow is in proportion to the degree of path coefficients. Green and red arrows indicate positive and negative relationships, respectively. *R*^2^ values indicate the proportion of variance explained by each variable. Significance levels are denoted with **P < 0.01 and ***P < 0.001. Standardized total effects (direct plus indirect effects) calculated by the SEM are displayed below the SEM. The low chi-square (χχ^2^), nonsignificant non-significant probability level (*P* > 0.05), high goodness-of-fit index (GFI > 0.90), low Akaike information criteria (AIC), and low root-mean-square errors of approximation (RMSEA < 0.05) listed below the SEMs indicate that our data matches the hypothetical models.

The correlation heatmap showed that the relationship between bacterial phyla and plant properties or environmental factors differed for the phyllosphere and rhizosphere (Figure 5). For the phyllosphere, the phyla *Actinobacteria* and *Bacteroidetes* exhibited significant positive correlations to the C/N of leaves, sheaths, and roots. Moreover, *Actinobacteria*, *Bacteroidetes*, and *Planctomycetes* exhibited extremely significant negative correlations to the TN of leaves and sheaths (Figure 5A). For the rhizosphere bacterial community, the abundance of *Proteobacteria* is extremely positive correlated to SWC, salinity, and EC, and significant negative correlated to the C/N of soil (Figure 5B). Soil properties and aboveground prosperities were the prominent factors influencing phyllosphere bacterial diversity. Also, root properties affected phyllosphere bacterial diversity, while the impacts of the plant development processes on the rhizosphere bacterial community were not observed (Figure 6).

**FIGURE 7:**
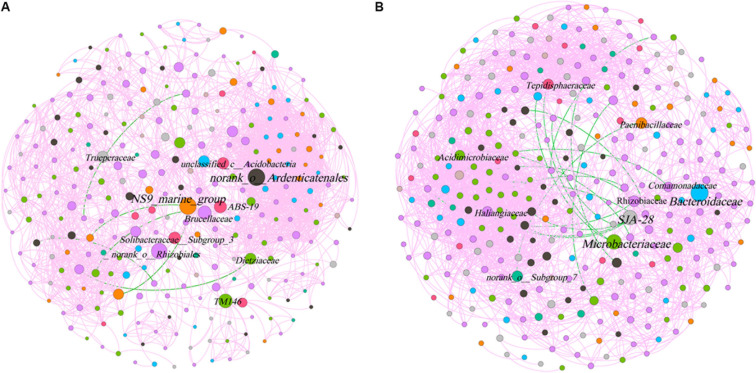
Co-occurrence network of microbial taxa on phyllosphere **(A)** and rhizosphere **(B)**. Nodes represent bacteria families, whereas pink and green edges, respectively, represent positive and negative connections between pairs of species.

The phyllosphere bacterial network in this study consisted of 282 nodes and 2,319 edges, and the network diameter and density were 10 and 0.059, respectively (Figure 7). The rhizosphere bacterial network consisted of 315 nodes and 2,845 edges, and the network diameter and density were 7 and 0.057, respectively. Both indicated that the coexistence among bacterial communities was greater than their exclusion in the phyllosphere (99.74%) and the rhizosphere (99.4%). *Bacteroidetes*, *Chloroflexi*, and *Proteobacteria* were the key bacterial phyla in the phyllosphere, while *Actinobacteria*, *Bacteroidetes*, and *Chlorobi* played critical roles in the rhizosphere (Table 3). *Bacteroidaceae* and *Microbacteriaceae* yielded the highest betweenness centrality (BC) values within the rhizosphere bacterial network, while they were also critical in maintaining the structure and function of the bacterial communities (Figure 7).

**TABLE 3:** Dominant keystone species (top 10) from phyllosphere and rhizosphere bacterial community structures.

	**Phylum**	**Family**	**Betweenness centrality**
Phyllosphere	*Acidobacteria*	*ABS-19*	1684.51
	*Acidobacteria*	*Solibacteraceae__ Subgroup_3_*	1603.16
	*Acidobacteria*	*unclassified_c__ Acidobacteria*	1539.13
	*Actinobacteria*	*TM146*	1977.28
	*Actinobacteria*	*Dietziaceae*	1465.58
	*Bacteroidetes*	*NS9_marine_group*	2518.01
	*Chloroflexi*	*norank_o__ Ardenticatenales*	2572.61
	*Deinococcus- Thermus*	*Trueperaceae*	1508.75
	*Proteobacteria*	*norank_o__Rhizobiales*	2339.50
	*Proteobacteria*	*Brucellaceae*	1893.29
Rhizosphere	*Acidobacteria*	*norank_o__Subgroup_7*	1733.79
	*Actinobacteria*	*Microbacteriaceae*	2923.67
	*Actinobacteria*	*Acidimicrobiaceae*	1474.28
	*Bacteroidetes*	*Bacteroidaceae*	3683.57
	*Chlorobi*	*SJA-28*	2845.07
	*Firmicutes*	*Paenibacillaceae*	1827.65
	*Planctomycetes*	*Tepidisphaeraceae*	1593.19
	*Proteobacteria*	*Rhizobiaceae*	1776.13
	*Proteobacteria*	*Haliangiaceae*	1498.62
	*Proteobacteria*	*Comamonadaceae*	1411.49

### Functional Features of Phyllosphere and Rhizosphere Bacterial Communities

We used the PICRUSt^10^ program to better understand the important role that bacteria play in the phyllosphere and rhizosphere, and we obtained a microbial COG profile and analyzed functional microbial features during three plant development stages (Supplementary Figure S4). The microbiomes exhibited distinct functional features associated with phyllosphere and rhizosphere bacterial communities. These functional features included amino acid transport and its metabolic processes, energy production and conversion, transcription, nucleotide transport and its metabolic processes, and carbohydrate transport and its metabolic processes (Supplementary Figure S4).

## Discussion

This study investigated the dynamic relationship of phyllosphere and rhizosphere bacterial communities during the plant development. The relative abundance and Shannon indices showed that rhizosphere bacterial communities during the seedling and tiller stages were significantly more diverse than those during the mature stage, while changes in the Simpson index had the opposite effect (Figure 1). These results agreed with previous studies, namely, that the rhizosphere microbial community of *Arabidopsis* differed significantly during the seedling stage compared to the other two stages (Chaparro et al., 2014). There were no significant differences in the relative abundance and diversity of the phyllosphere bacterial community during these three stages (Figure 1). Similarly, we observed no distinguishable trends in patterns of change in bacterial diversity during plant growth stages, and this is consistent with a report from a previous study (Redford and Fierer, 2009), demonstrating that there were no discernible patterns of change in phyllosphere bacterial diversity. For phyllosphere microbiomes of trees or perennial plant species, less migration from soil could simply be the result of distance or the longer time required for the host to adapt (Kim et al., 2012).

The dominant phyllosphere bacterial phyla varied among the plant growth stages, but the dominant bacteria during the three plant development stages were similar in the rhizosphere (Supplementary Figure S2). This was consistent with results from earlier studies on other plant species deduced from pyrosequencing, such as lettuce (Rastogi et al., 2012), fresh spinach (Lopezvelasco et al., 2011), and tree species (Redford et al., 2010). The role of *Bacteroidetes* in the phyllosphere has yet to be clarified although one study has indicated that it is critical to nutrient turnover (Yousuf et al., 2012). Moreover, bacterial species that belong to the *Bacteroidetes* phylum comprise of genes associated with denitrification, suggesting a potential role in N cycling (Spanning et al., 2005). During the seedling stage, *Cyanobacteria* and *Actinobacteria* showed different dynamic change trends. Recently, *Actinobacteria* has been correlated to disease suppressive soils (Rodrigo et al., 2011). Moreover, *Cyanobacteria* is known to colonize plant roots (Gantar et al., 2010; Lundberg et al., 2012), which can encourage plant growth (Prasanna et al., 2009). Meanwhile, *Cyanobacteria*, owing to its N-fixation ability, are a key source of inorganic N for plants (Franche et al., 2009). In future studies, *Cyanobacteria* and *Actinobacteria* could be inoculated in the process of bioremediation, and studied for their effects on plant growth and physiology, so as to better restore contaminated sites via a combined microbial and plant interaction.

The predominant bacterial components of samples during the plant development stages were similar to that of the rhizosphere (Supplementary Figure S2B). In contrast to previous studies, our more detailed examination of rhizosphere microbial communities during plant development stages revealed the formation of a central microbiome composed of bacteria from various phyla (*Actinobacteria*, *Bacteroidetes*, *Proteobacteria*, *Chloroflexi*, *Firmicutes*, *Gemmatimonadetes*, *Nitrospirae*, *Planctomycetes*, and *Verrucomicrobia*) (Bulgarelli et al., 2012; Lundberg et al., 2012), and *Actinobacteria* was observed to significantly change during plant development (Chaparro et al., 2014). *Proteobacteria* was the prevailing bacterial phylum found in both the phyllosphere and rhizosphere, which indicated that members of this phylum could be involved in both the physiological and the ecological development of *B. ischaemum* in contaminated environments. One study reported that *Proteobacteria* was the most stress-tolerant phylum under conditions of heavy soil contamination (Eva-Maria et al., 2011). It has also been suggested that numerous heavy metal oxidase genes encoded by *Proteobacteria* resist heavy metal contamination (Altimira, 2012). Futhermore, *Proteobacteria* has previously exhibited considerable diversity in morphology, physiology, and metabolic processes (Zavarzin et al., 1991), suggesting that this phylum can adapt to different phyllosphere environments by physiological and metabolic regulation. For the rhizosphere bacterial community, *Proteobacteria* and *Acidobacteria* exhibited significant differences at a phylum level (Figure 3). It is accepted among researchers that plant species can adopt a microbial subset at various growth stages. The role of *Acidobacteria*, an abundant bacterial phylum (Barns et al., 1999), is important in C cycling, because of its capacity in degrading complex polysaccharides, such as cellulose and lignin, derived from plants (Ward et al., 2009). Moreover, these results reinforced findings from previous studies that reported that temporal variability in phyllosphere and rhizosphere bacterial community composition was not random. During the different growth stages, variation in the structure and composition of bacterial communities can be explained to some extent by differences in abiotic environments, including climate conditions and plant and soil chemical characteristics.

During the three plant stages, phyllosphere and rhizosphere bacterial communities separately correlated to plant and soil chemical characteristics (Figure 4). Phyllosphere bacterial community structure was significantly affected by plant N, which implied that the effects of N on the bacterial community were variable and likely stage dependent (Fierer et al., 2012). The structure of microbiota is typically shaped by abiotic and biotic environmental variables. Soil properties, aboveground and root prosperities were the prominent factors influencing phyllosphere bacterial diversity (Figure 6). Moreover, taxa that can take advantage of a variety or a specific subset of carbohydrates will thrive in the phyllosphere as indicated by the enrichment of carbohydrate transport and the metabolism of gene families. This could be caused by environmental conditions under which *B. ischaemum* is grown, namely, geographic characteristics and climatic parameters. It has been reported that *Enterobacter* had a negative impact on the *Erwinia* bacteria group during phyllosphere colonization (Hunter et al., 2010).

Keystone microbes can be generally defined as those species that have a disproportionate influence on ecosystems regardless of abundance, and they are crucial in the maintenance of the stability and the function of ecosystems as well as the resistance of system disturbances (Lupatini et al., 2014; Zhang et al., 2018). Some keystone families were identified from co-occurrence network, demonstrating the dynamics of the relationships between phyllosphere and rhizosphere bacterial communities during different stages of plant development (Figure 7). Moreover, it has been confirmed that certain taxa of low abundance paradoxically play a disproportionate role in regulating ecological functions in various habitats (Zhang et al., 2018). Keystone microbes exhibited significant differences between phyllosphere and rhizosphere bacterial communities. The key to comprehending the development of bacterial leaf communities in environments under stress is to understand the symbiotic relationships that exist between microbes that have successfully adapted to their host plants as well as plant health development (Andrews, 1992).

It is important to note that any alteration to either bacterial community structure or composition will likely have functional consequences (Peñuelas et al., 2012). Furthermore, enrichment in metabolic functions was observed in the phyllosphere and rhizosphere, which implied that microbial (bacterial) metabolism tended to be vigorous. The role of both phyllosphere and rhizosphere microbiomes are vital to plant productivity and health, being also referred to as the “second” genome of plant species (Berendsen et al., 2012; Chaparro et al., 2014). The phyllosphere and rhizosphere bacterial communities had distinct functional features. This indicated that phyllosphere and rhizosphere microbial communities select specific functions throughout plant development (Chaparro et al., 2014). These results shed new light on understanding the dynamic relationship of phyllosphere and rhizosphere bacterial communities during the development of *B. ischaemum* in a pollution environment. They are beneficial to the development and utilization of beneficial microbial communities at different stages of development, which might help to reclaim and stabilize tailings more effectively.

## Data Availability Statement

The bacterial sequences have been deposited in the SRA of the NCBI database under the SRA accession: PRJNA600330.

## Author Contributions

TJ conceived and designed the experiments. RW and YY performed the experiments. BC contributed new reagents. TJ and TW wrote the manuscript. All authors read and approved the manuscript.

## Conflict of Interest

The authors declare that the research was conducted in the absence of any commercial or financial relationships that could be construed as a potential conflict of interest.
