# Supplementary material for: Dynamics Relationship of Phyllosphere and Rhizosphere Bacterial Communities During the Development of Bothriochloa ischaemum in Copper Tailings
Source: Front Microbiol. 2020 May 28;11:869. doi: 10.3389/fmicb.2020.00869 (PMC7270166; doi:10.3389/fmicb.2020.00869)
Supplement: Supplementary file 1 [file Data_Sheet_1.PDF]

Dynamics relationship of phyllosphere and rhizosphere bacterial communities during the development of *Bothriochloa ischaemum* in a copper tailings

Tong Jia[1]*, Yushan Yao[1], Ruihong Wang[1], Tiehang Wu[2], Baofeng Chai[1]

[1]Shanxi Key Laboratory of Ecological Restoration on Loess Plateau, Institute of Loess Plateau, Shanxi University, Taiyuan 030006, P.R. China;

[2]Department of Biology, Georgia Southern University, Statesboro, GA 30460-8042, USA

*Corresponding author. Email: jiatong@sxu.edu.cn

Address: No. 92 Wucheng Road, Xiaodian district, Taiyuan, Shanxi 030006, P.R. China. Tel.: +86-155-1369-4458

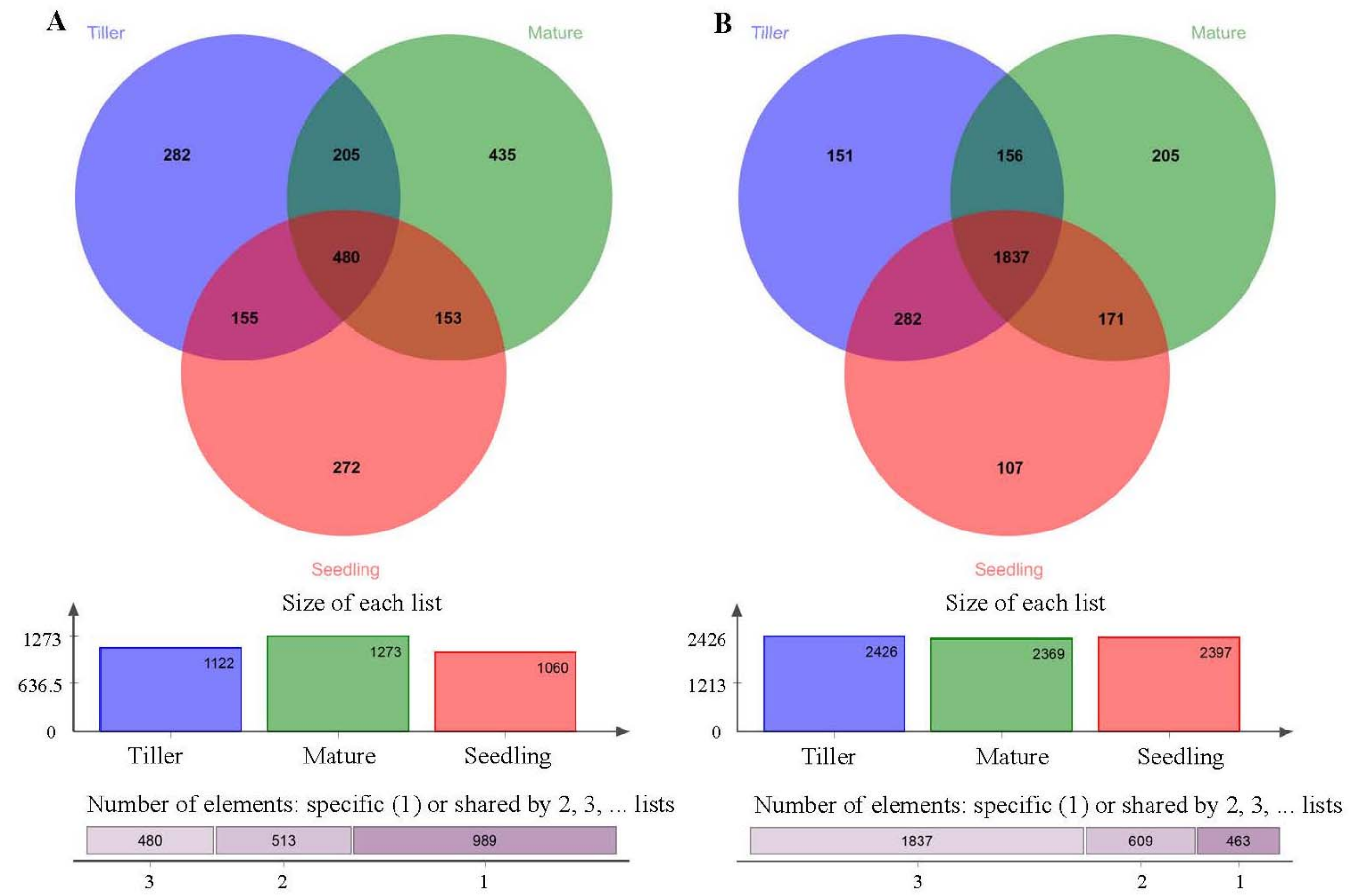

FIGURE S1 Venn diagram for phyllosphere (A) and rhizosphere (B) bacterial communities among plant development processes. Numbers indicated shared unique operational taxonomic units (OTUs) at 0.03 dissimilarity distances after removing singletons involved.

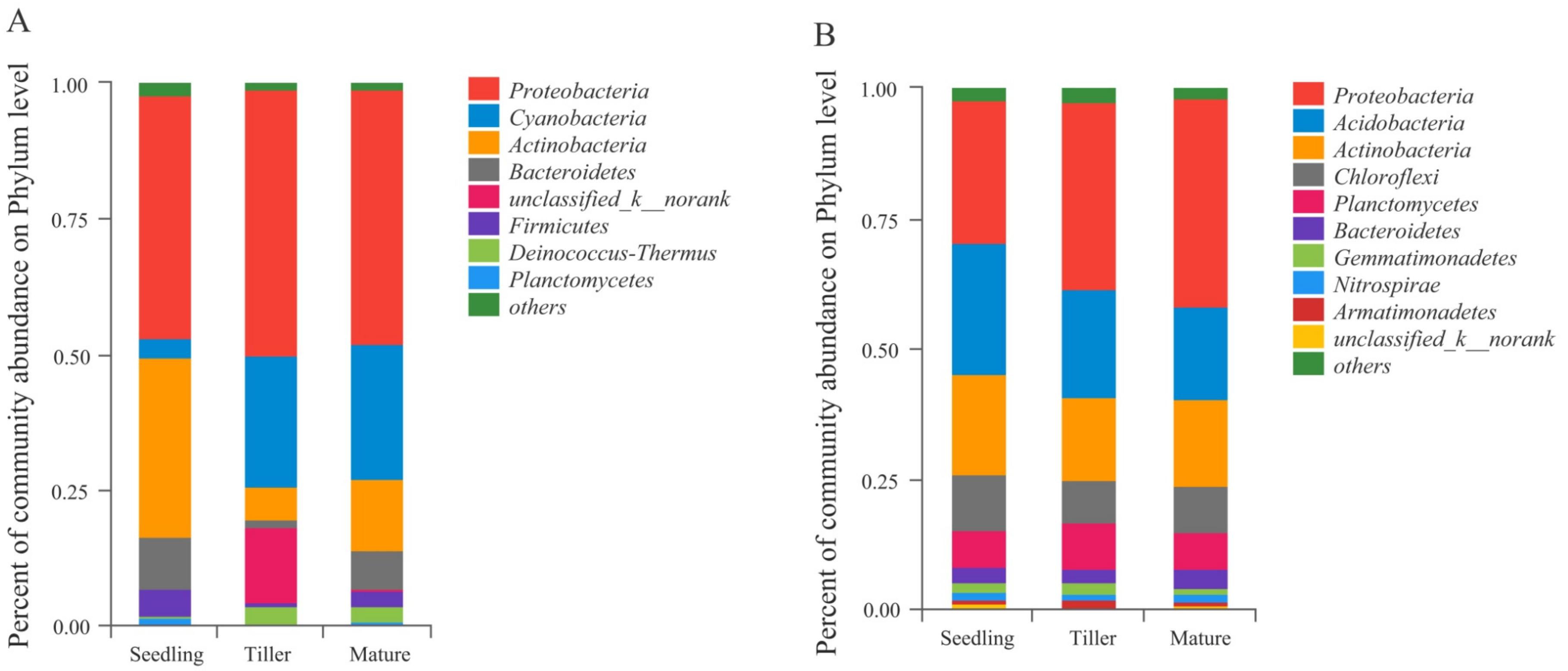

FIGURE S2 Relative abundance of the dominant bacterial phyla in phyllosphere (A) and rhizosphere (B) samples (with average relative abundance > 1%) among different plant growth stages.

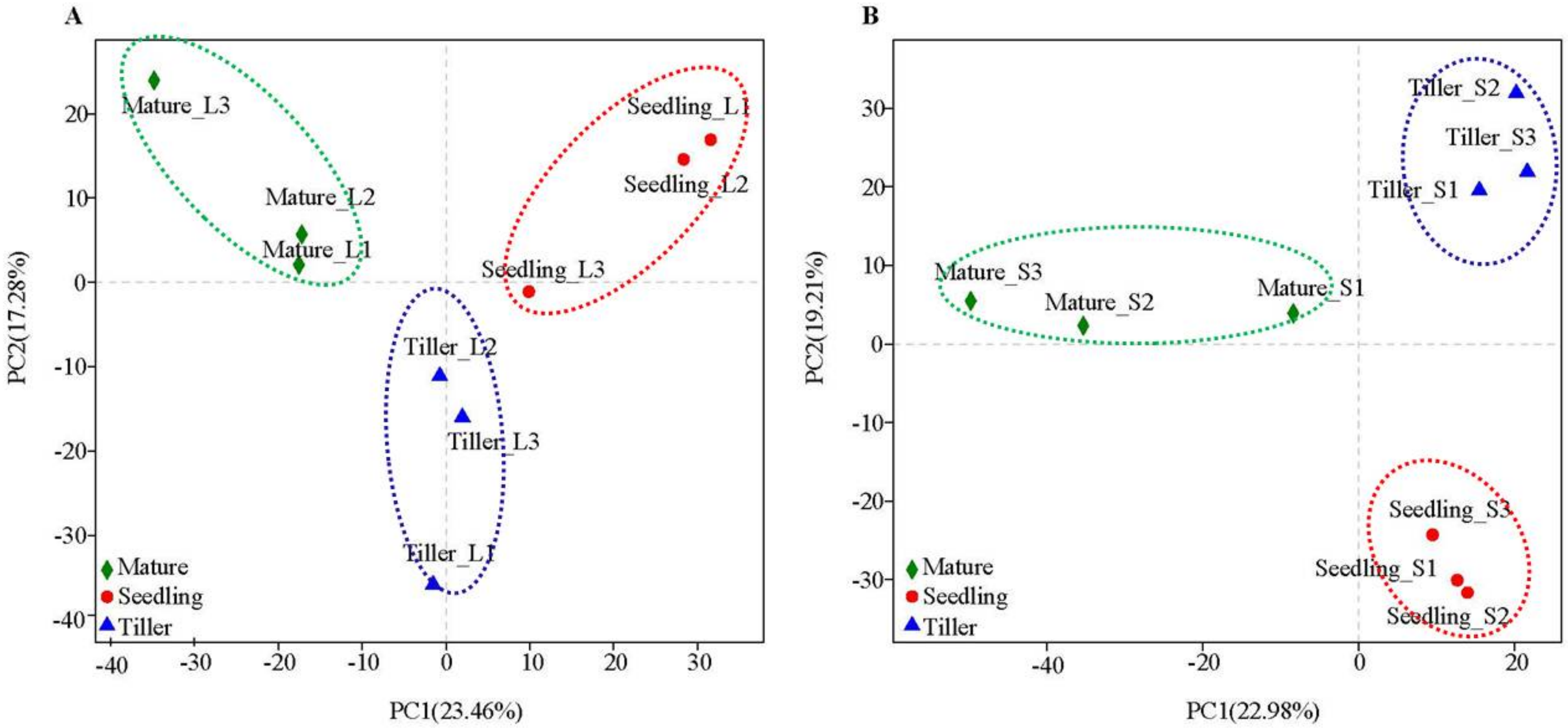

FIGURE S3 Principal component analysis (PCA) of phyllosphere (A) and rhizosphere (B) bacterial communities based on Bray-Curtis distance indices and operational taxonomic units (OTUs) at three different plant growth stages. The dashed ovals indicate a significant influence of plant development on bacterial community structure (assessed by a multivariate analysis of similarities (ANOSIM)).

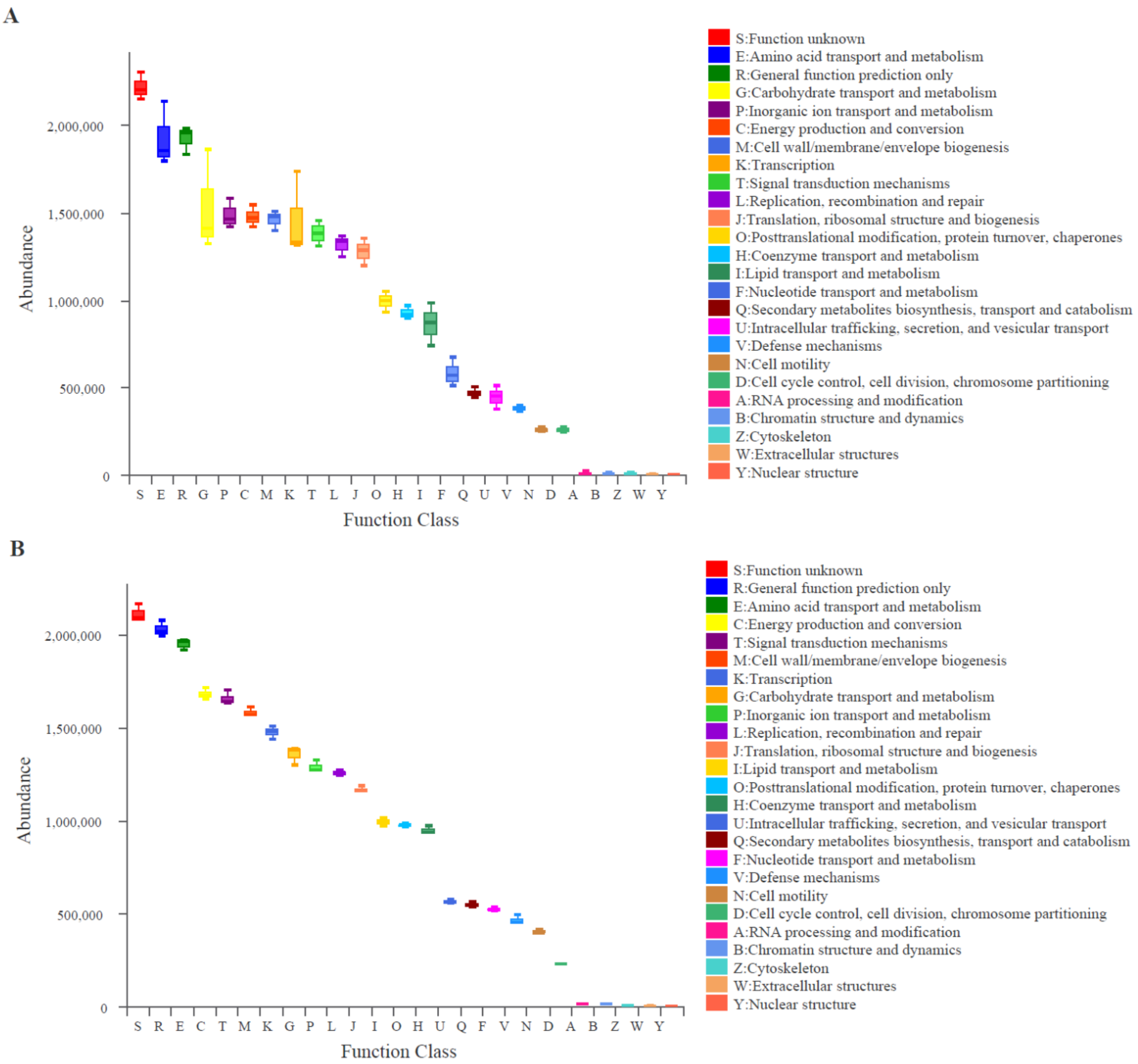

FIGURE S4 Functional features of phyllosphere (A) and rhizosphere (B) bacterial communities in copper tailings dam.
